# The NanoSuit method: a novel histological approach for examining paraffin sections in a nondestructive manner by correlative light and electron microscopy

**DOI:** 10.1038/s41374-019-0309-7

**Published:** 2019-08-29

**Authors:** Hideya Kawasaki, Toshiya Itoh, Yasuharu Takaku, Hiroshi Suzuki, Isao Kosugi, Shiori Meguro, Toshihide Iwashita, Takahiko Hariyama

**Affiliations:** 1 Institute for NanoSuit Research, Preeminent Medical Photonics Education & Research Center, Hamamatsu University School of Medicine, Hamamatsu, Japan; 2 Department of Obstetrics and Gynecology, Hamamatsu University School of Medicine, Hamamatsu, Japan; 3 Department of Chemistry, Hamamatsu University School of Medicine, Hamamatsu, Japan; 4 Department of Regenerative and Infectious Pathology, Hamamatsu University School of Medicine, Hamamatsu, Japan

**Keywords:** Electron microscopy, Diseases

## Abstract

Histological examination using the light microscopy is currently the gold standard for life science research and diagnostics. However, magnified observations are limited because of the limitations intrinsic to light microscopy. Thus, a dual approach, known as correlative light and electron microscopy (CLEM), has emerged, although several technical challenges remain in terms of observing myriad stored paraffin sections. Previously, we developed the NanoSuit method, which enabled us to keep multicellular organisms alive/wet in the high vacuum of a scanning electron microscope by encasing the sample in a thin, vacuum-proof membrane. The approach uses the native extracellular substance (ECS) or an ECS-mimicking substance to polymerize a membrane by plasma or electron beam irradiation. Since the resulting NanoSuit is flexible and dense enough to prevent a living organism’s bodily gas and liquids from evaporating (which we refer to as the “surface shield enhancer” (SSE) effect), it works like a miniature spacesuit with sufficient electron conductivity for an SEM observation. Here, we apply the NanoSuit method to CLEM analysis of paraffin sections. Accordingly, the NanoSuit method permits the study of paraffin sections using CLEM at low and high magnification, with the following features: (i) the integrity of the glass slide is maintained, (ii) three-dimensional microstructures of tissue and pathogens are visualized, (iii) nuclei and 3,3′-diaminobenzidine-stained areas are distinct because of gold chloride usage, (iv) immunohistochemical staining is quantitative, and (v) contained elements can be analyzed. Removal of the SSE solution after observation is a further advantage, as this allows slides to be restained and stored. Thus, the NanoSuit method represents a novel approach that will advance the field of histology.

## Abstract (web summary)

The NanoSuit method permits the study of paraffin sections using correlative light and electron microscopy at low and high magnification, with the following features: (i) the integrity of the glass slide is maintained, (ii) 3D microstructures of tissue and pathogens can be visualized, (iii) nuclei and 3,3′-diaminobenzidine-stained areas are distinct because of gold chloride usage, (iv) immunohistochemical staining is quantitative, and (v) contained elements can be analyzed.

## Introduction

While histological examination using light microscopy is the gold standard for life science research, it has limitations, including low resolution (a few hundred nanometers) caused by the light-diffraction limit and images being limited to two-dimensions owing to the small focal depth even for thin sections (4–6 µm). To compensate for those limitations, we developed a method to observe sections on glass slides for light microscopy with an electron microscope. Developing a convenient and accurate correlative observation method between light and electron microscopy could facilitate a combination of their respective advantages.

Sections of formalin-fixed paraffin-embedded (FFPE) tissues with hematoxylin and eosin (H&E) staining have been used to diagnose many diseases, and to evaluate morphological structures and pathological changes for over a century. Immunohistochemical (IHC) staining of sections is also used to investigate the cellular functions of proteins and for diagnostic pathological applications. Sections mounted on a glass sglide with a coverslip represent the most common method of storing clinical tissue specimens. The great advantage of light microscopy is that objects with natural or stained colors can be examined with little or no additional treatment. Therefore, screening for pathological lesions is simple and fast. In addition to this technical convenience, a substantial body of data has been accumulated using this method during its long history. Thus, we chose to compare the correlative area of the glass slide using light and electron microscopy.

Recently, it has become necessary to use high magnifications to analyze specific target sites for diagnostic purposes, especially for kidney pathological lesion [1]. Given the need for higher magnification and resolution, scanning electron microscope (SEM)-based methods have been developed for working with FFPE sections [1–3]. Because SEM images can also be observed stereoscopically, Sawaguchi et al. developed a three-dimensional (3D) survey method for assessing cell/tissue architectures in 30-µm-thick paraffin sections in a low-vacuum scanning electron microscope (Lv-SEM) [3] in combination with a heavy metal coating, which increased both the image contrast and electron conductivity. In general, SEM requires preparation procedures for insulating biological samples and glass slides, which involve sputtering with conductive materials, such as carbon, gold, platinum, palladium, or alloy to prevent charging effects. Inaga et al. reported that Lv-SEM examination with platinum blue was a simple and useful method for directly observing histological paraffin-embedded kidney sections [1]. These preparatory procedures may cause some observational artifacts and prevent the restoration of the original specimens. Thus, it is necessary to develop a method for observing specimens that can be easily stored without complicated preparation procedures, which readily decrease sample features.

Unfortunately, an SEM capable of high resolution can only capture black and white images. The ideal combination for observing a region of interest would involve the ability to screen and extract colored images using various types of light microscopes, and subsequently, observe 3D images of the same region at high resolution using SEM. Several reports have been published on the subject of correlative microscopic examinations with paraffin sections [3–5]. However, this is complicated by the difficulty in finding the region of interest observed by light microscope again with SEM. To overcome this challenge, correlative light and electron microscopy (CLEM) equipment has been developed and has been in general use for several years [6, 7]. However, it remains a costly method with complicated sample preparation. Given that each piece of CLEM equipment requires a special jig device to define the location, it is currently impossible to utilize paraffin sections on conventional glass slides.

Previously, we developed an advanced method for observing 3D structures of wet organisms by SEM, which we named the “NanoSuit method.” Using this method, multicellular organisms can be kept alive/wet under a high vacuum within an electron microscope by encasing them in a thin, vacuum-proof membrane [8]. The approach uses the native extracellular substance (ECS) or an ECS-mimicking substance to polymerize a membrane by plasma or electron beam irradiation. Since the resulting NanoSuit is flexible and dense enough to prevent the living organism’s bodily gas and liquids from evaporating, which we refer to as the “surface shield enhancer” (SSE) effect, it works like a miniature spacesuit with sufficient electron conductivity for an SEM observation. Thus, the NanoSuit method enables high-resolution imaging of wet specimens, both living and fixed, by an SEM [9].

Here, we applied the NanoSuit method to CLEM for paraffin sections using whole glass slides. This application enabled the versatile study of the same structures by CLEM to observe tissue and pathogens with several analytical methods in an SEM without destruction. Therefore, the NanoSuit method will be useful as a salvage/recovery technique.

## Materials and methods

### Sample selection

The use of human tissue samples and pathological specimens was approved by the Research Ethics Board at Hamamatsu University School of Medicine (approval number 18-074).

### H&E staining of paraffin sections

FFPE tissue sections, ~4 μm thick, were deparaffinized in xylene and hydrated using serial percentages of alcohol. The sections were stained with hematoxylin for 20 min and with eosin for 10 min The cover glass was mounted with diaphane (i.e., a mounting medium for sealing a cover glass on a glass slide) (Malinol, Muto Pure Chemicals Co., LTD, Tokyo, Japan).

### IHC staining of paraffin sections

IHC staining of paraffin sections was performed using antibodies directed against smooth muscle actin (monoclonal antibody; DAKO, Tokyo, Japan), human epidermal growth factor receptor 2 (HER2) (monoclonal antibody; Nichirei Bioscience), CD1a (monoclonal antibody; Leica, Newcastle, UK), the Cytomegalovirus glycoprotein B (CMV-gB) protein (biotin-conjugated polyclonal antibody; Bioss, Inc., Massachusetts, USA), or F-actin (biotin-conjugated polyclonal antibody; Bioss, Inc.) for 30 min at room temperature (20−30 °C). Those antibodies required antigen retrieval procedures using citrate buffer with heating at 95 °C, proteinase K digestion at room temperature, or treatment with L.A.B. solution (Polysciences, Inc. PA, USA) at room temperature. After washing in phosphate-buffered solution (PBS; 137 mM NaCl, 10 mM phosphate, 2.7 mM KCl, pH 7.4), the sections were incubated with a peroxidase- or biotin-conjugated universal immunoenzyme polymer anti-mouse or anti-rabbit solution (Nichirei Biosciences) and visualized using streptavidin-conjugated gold (40 nm; Abcam, Cambridge, UK) or 3,3′-diaminobenzidine (DAB) (DAKO). After washing the section three times with PBS containing 0.1% Triton X, it was completely covered with 100–200 μl DAB-substrate and incubated for 10 min at room temperature.

IHC was used to show whether cancer cells express HER2 receptors and/or hormone receptors on the surface information that is critical for treatment planning. The IHC test gives a score of 0 to 3+, depending on the amount of HER2 receptor protein on the surface of cells in a breast cancer tissue sample [10]. Breast cancer sections with a HER2 score of 3+ and a HER2 score of 0 were compared. For SEM observations, paraffin sections were exposed to an anti-HER2 antibody. After washing, the sample was treated with a biotin-conjugated secondary antibody and streptavidin-conjugated 40-nm gold particles.

### Sample preparation for SEM

**Fig. 1:**
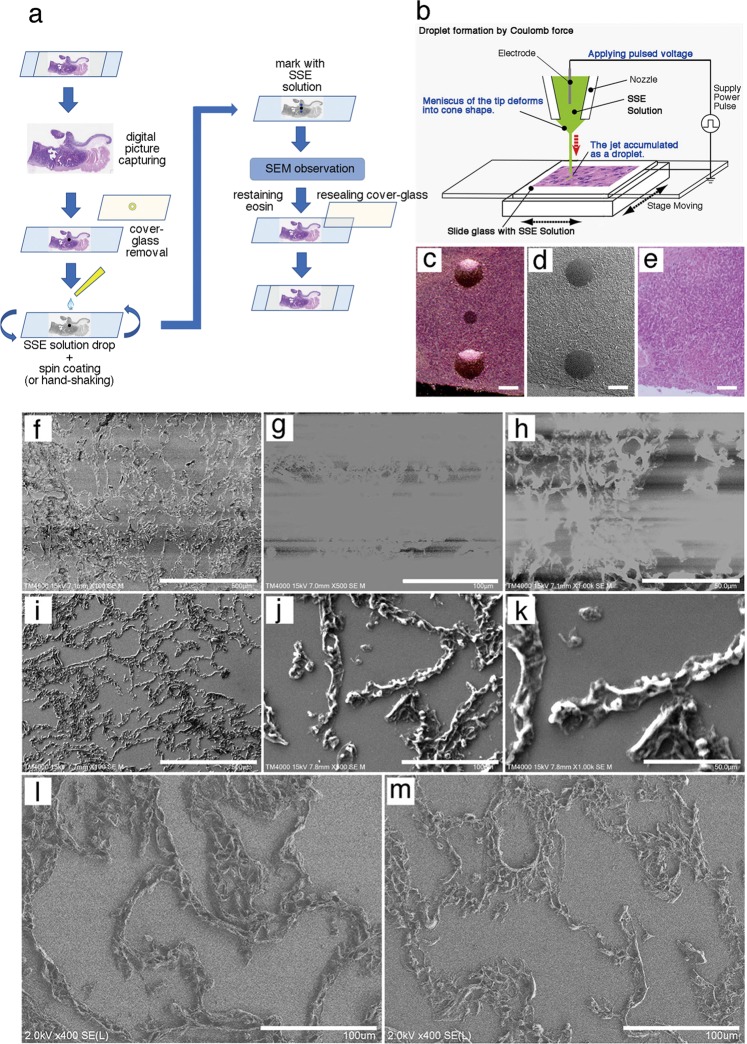
NanoSuit method for observing paraffin sections by scanning electron microscopy (SEM). **a** Workflow for correlative microscopy analysis of paraffin sections with the NanoSuit method. **b** On-demand droplet spotter system used to place marks around the region of interest using surface shield enhancer (SSE) solution. Droplets of undiluted SSE stock solution were identified as markers around the region of interest (black mark) and imaged using a light microscope (**c**) and a low-vacuum scanning electron microscopy (Lv-SEM) (**d**). **e** Image of a tissue section restained with hematoxylin and eosin after SEM observation and removing the SSE solution markings. The white bars shown in **c–e** represent 1 mm. **f**–**h** High charging effects of control sections without SSE solution (Lv-SEM in the secondary electron image). **i**–**k** SSE solution-coated sections showing a reduced charging effect (Lv-SEM in the secondary electron image). **l**, **m** field emission-scanning electron microscope (FE-SEM) secondary electron image of lung alveolar tissue. **l** NanoSuit-coated lung tissue. **m** Carbon-coated lung tissue. The scale bars represent 500 (**f**, **i**), 100 (**g**, **j**, **l**, **m**), and 50 μm (**h**, **k**)

The preparation of specimens for correlative observations with the NanoSuit method is summarized in Fig. 1a. H&E-stained and immunostained sections were observed with a light microscope. The region of interest was marked with a circle using a water/organic solvent-resistant pen (Frost Marker, Matsunami Glass Ind., Ltd, Kishiwada, Japan), both on the front of the coverslip and on the back of the glass slides. Marked sections were captured with a NanoZoomer-RS digital camera (Hamamatsu Photonics Co. Hamamatsu, Japan) or other digital cameras. Digital images could be observed on the computer screen while using an SEM. The marked specimens were incubated in xylene for 15–20 h at room temperature to remove the coverslips. The sections were rehydrated with a series of 100, 90, and 70% ethanol and distilled water (5 min each), and then the SSE liquid (NanoSuit solution) was applied to the surface of the specimen on the glass slides. After SEM observation, NanoSuit-coated and marked sections were restained with eosin for 10 min and dehydrated with a series of 70, 90, and 100% ethanol and xylene. The glass coverslip was remounted with diaphane.

### Application of the NanoSuit method to paraffin sections

The newly developed SSE [9] was used in all experiments. SSE stock solution consisted of sucrose, fructose, and sodium chloride dissolved in distilled water, to which citric acid and sodium glutamate (pH 7.4) were then added. The resulting aqueous solution was mixed with glycerin at a ratio of 1:2. SSE was diluted 20-fold in water or ethanol, depending on the experimental design. After taking digital image information, a tissue section containing a region of interest is marked on both the front of the coverslip and the back of the microscope slide using a water/organic solvent-resistant pen (Fig. 1a), the mark on the back of the slide serves as a guiding point for subsequent marking with SSE solution. Subsequently, diaphane and coverslip were removed from the slide glass, and a diluted NanoSuit liquid (SSE) solution was applied to the surface of the tissue section, covering the whole glass slide and allowed to stand for 1 min Excess SSE solution was removed by spin coating (2000 rpm, 15 s) (or hand shaking the glass slide strongly) (Fig. 1a).

### On-demand droplet spotter

Marking around the designated pathological site was performed using the on-demand droplet spotter (Hamamatsu Nanotechnology Co. Hamamatsu, Japan). The on-demand Droplet Spotter system can dispense a minute volume (pL–fL) of solution and is applicable to high-viscosity solutions such as SSE liquid. When a pulse voltage was applied between liquid and a substrate, the jet was ejected from the apex of the glass capillary. The ejected liquid accumulated as a droplet. Minute drops of SSE stock solution are applied at multiple precise locations with the computer-controlled droplet spotter system above the black mark on the slide (Fig. 1b). The coated SSE stabilized the surface electronic potential of the glass slide to produce stable droplets. Specimens were directly introduced into an SEM, and then the NanoSuit was formed following irradiation with the electron beam.

### Gold chloride solution application on H&E- and DAB-stained sections

For nuclear staining or DAB enhancement of SEM observations, H&E sections were incubated with 0.001–1% gold (III) chloride solution (diluted in water) for 5–10 min at room temperature and subsequently washed five times with water. SSE liquid was applied to each section.

### Low-Vacuum SEM (Lv-SEM) image acquisition

The glass slide was covered with SSE solution and set onto the wide stage of the specimen holder using adhesive conductive tape (Nisshin EM Co. Ltd, Japan) and then placed in an Lv-SEM (TM4000Plus, Hitachi High-Technologies, Tokyo, Japan). After the evacuation of the specimen chamber for a few minutes, the area of the specimen marked with SSE solution droplets was observed under the electron beam at an accelerating voltage of 5, 10, or 15 kV. Lv-SEM images were taken using the secondary electron (SE), backscattered electron (BSE), and energy-dispersive spectrometry (EDS) detectors.

### Field emission (FE)-SEM microscopy

FE-SEM was performed with a Hitachi S-4800 instrument operated at an acceleration voltage of 1.0–5.0 kV. The vacuum level of the observation chamber ranged from ~10^−3^ to 10^−6^ Pa. Detection of SEs was achieved using a detector placed at the lower position. The BSE image was taken using an Yttrium–Aluminum–Garnet (YAG) BSE detector. Further experimental parameters were as follows: working distance: 8 mm; aperture size: ϕ 100 μm; scan speed: 10–15 frames/s.

### Energy-dispersive X-ray spectrometry (EDX) analysis of paraffin sections

Scanning electron microscopy combined with energy-dispersive X-ray spectrometry (EDX) is a spectral technique that provides visual identification of multiple elements simultaneously. To analyze the elemental components of paraffin sections by SEM/EDX, the procedure described in Fig. 1 was performed to observe identical H&E specimens. A built-in EDX detector (manufactured by Hitachi High-Technologies Corporation of Oxford Instruments Analytical Ltd, UK) was used with the Lv-SEM (TM4000Plus). Elemental analysis was performed with paraffin sections coated with SSE solution, using a 15 kV electron beam.

## Results

### Preparation of paraffin sections for correlative observations with the NanoSuit method

The preparation of specimens for correlative observations with the NanoSuit method is summarized in Fig. 1a. Because SSE solution droplets made by droplet spotter system (Fig. 1b) can be identified both by light microscopy (Fig. 1c) and SEM (Fig. 1d), the spotted droplets served as a marker to identify the region of interest. Importantly, this marking procedure did not cause any mechanical damage to the section on the slide. Spotted marking (Fig. 1d) could be removed completely while re-staining with the original staining color to restore the slide (Fig. 1a, e). These results ensure that conventional light microscope sections can easily be subjected to correlative observations without causing damage.

The sections of lung alveolar walls were used to evaluate the applicability of the NanoSuit method for SEM observations. In the secondary Lv-SEM image obtained with a high voltage beam (15 kV), the addition of a small amount of SSE (tenfold dilution in ethanol) notably prevented charging effects (Fig. 1i–k) compared with nontreated sections (Fig. 1f–h). Our FE-SEM showed clearer images of paraffin sections than did the Lv-SEM (Fig. 1l, m). The NanoSuit-coated sample (Fig. 1l) was clearly thicker than the carbon-coated sample (Fig. 1m).

### Observing microorganisms in paraffin sections using the NanoSuit method

**Fig. 2:**
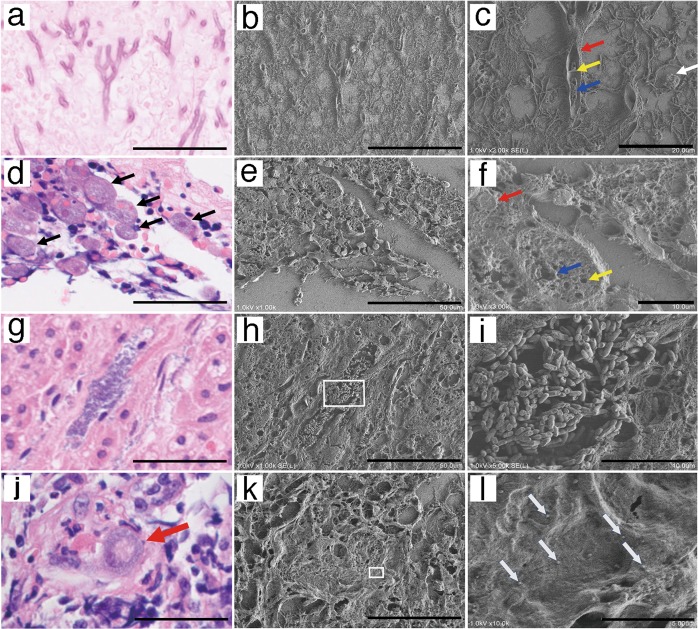
Application of the NanoSuit method to pathogens in paraffin sections. **a**–**c** Lung infection with *Aspergillus* **a** hematoxylin and eosin (H&E) image. **b** Identical field emission-scanning electron microscope (FE-SEM) secondary electron image. **c** Enlarged FE-SEM secondary electron image. Sporangium (white arrow), cross-sectional three-dimensional (3D) structure of *Aspergillus hyphae* including the cell wall (red arrow), septum (yellow allow), and nuclei (blue arrow). **d**–**f** Entamoeba histolytica infection of the colon. **d** H&E image. Multiple amoeba bodies (black arrows). **e** Identical FE-SEM secondary electron image. **f** Enlarged FE-SEM secondary electron image. Cross-sectional 3D structures of an amoeba body including vacuolar structures (contractile vacuole [blue arrow], food vacuole [yellow arrow]) in the cytoplasm and the nucleus (red arrow). **g**–**i** Bacterial infection. **g** H&E image. **h** Identical FE-SEM secondary electron image of **g**. **i** Magnified FE-SEM secondary electron image of **h** (white square). **j**–**l** Cytomegalovirus (CMV) infection of a kidney. **j** A cytomegalic inclusion body of an H&E section is shown (red arrow). **k** Identical FE-SEM secondary electron image. The white square is magnified in **l**. **l** Enlarged FE-SEM secondary electron image. Multiple human CMV-like particles adjacent to a cytomegalic inclusion body. The scale bars represent 50 (**a**, **b**, **d**, **e**, **g**, **h**, **j**, **k**), 20 (**c**), 10 (**f**, **i**), and 5 μm (**l**)

To observe microstructures within a cell, we chose pathological specimens containing pathogens such as a virus, bacterium, protozoa, fungus, or other microorganism, as many pathogens are too small to be identified by light microscopy. Accordingly, the same pathological lesion found in an H&E-stained paraffin section of a lung patient affected by *Aspergillus* was observed both by light microscopy (Fig. 2a) and FE-SEM with the NanoSuit method (Fig. 2b). Moreover, images at high magnifications showed no shrinkage or collapse (Fig. 2c). Obscure eosinophilic fibrinous structures neighboring *Aspergillus* (Fig. 2a) observed by light microscopy could be clearly visualized by SEM as 3D structures (Fig. 2b, c). A sporangium, cell wall, septum, and nucleus were clearly observed as cross-sectional 3D structures in the magnified image (Fig. 2c), which could not be identified using H&E staining.

Next, colon tissue was biopsied from a patient with severe diarrhea and diagnosed with amoebic dysentery. Multiple bodies of *Entamoeba histolytica* were found in an H&E section (Fig. 2d). When the same section was observed by NanoSuit (Fig. 2e), the cross-sectional 3D structure of an amoeba body, including contractile and food vacuoles in the cytoplasm and the nucleus were observed in higher magnification (Fig. 2f).

Given that bacteria are usually too small to be observed under light microscopy, microbial colonies in adrenal tissue are difficult to differentiate based on shape (Fig. 2g). In contrast, observation of the same region using the NanoSuit method enabled clear visualization of the speculative bacterial lesion as a 3D image showing each rod-shaped bacterium (Fig. 2h, i). The bacteria were subsequently identified as *Aeromonas hydrophila* by culture.

This method was also applied to viral pathogens in paraffin sections. Cytomegalovirus (CMV) is a widespread virus, with manifestations varying from asymptomatic to severe end-organ dysfunction in immunocompromised patients or those with congenital CMV disease. Figure 2j shows a light microscope image of a cytomegalic inclusion body from the kidney of the patient infected with CMV. Multiple human CMV-like particles (100–300 nm, Fig. 2l) were observed adjacent to the inclusion body (Fig. 2k white square).

### Identifying microstructures in paraffin sections using the NanoSuit method

**Fig. 3:**
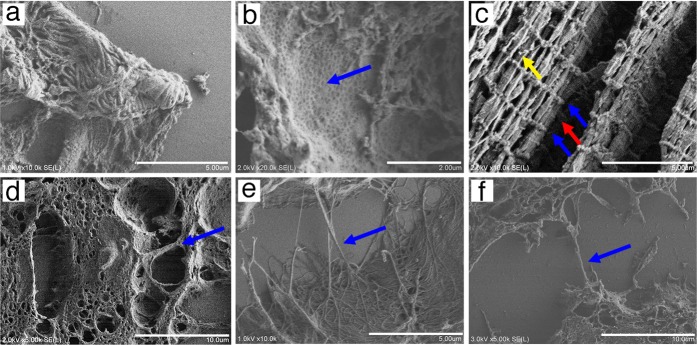
Microstructures of paraffin section tissues visualized by the NanoSuit method in field emission-scanning electron microscopy secondary electron images. **a** Kidney podocyte. **b** Inner aspect of fenestrated endothelium (blue arrow) of the glomerular capillary. **c** Skeletal muscle tissue. Striated pattern of myofibrils (Z lines within I-band [blue arrows] and M lines within an A-band [red arrow]) and spherical mitochondria (yellow arrow). **d** Projection structure in a malignant mesothelioma specimen (blue arrow). **e** Collagenous fibril in the perivascular area of colon tissue. **f** Connecting fibers between malignant breast cancer cells (tunneling nanotubes [TnT]-like structures). The scale bars represent 10 (**d**, **f**), 5 (**a**, **c**, **e**), and 2 μm (**b**)

The NanoSuit method was also applied to observe microstructures in paraffin sections of normal and malignant tissues. The structure of podocytes (Fig. 3a) and fenestrated capillaries (Fig. 3b) of the kidney were also shown using the NanoSuit method in high vacuo with FE-SEM. When skeletal muscle fibers were observed using the NanoSuit method, striated patterns of myofibrils (Z lines within I-bands and M lines within A-bands) and spherical mitochondria were visible as 3D structures in the longitudinal section (Fig. 3c). The 3D structure of the protrusions of mesothelioma cells (Fig. 3d) and collagenous fibrils (Fig. 3e) were also clearly visualized, as well as connective fibers mimicking tunneling nanotubes (TNTs) between cancer cells (Fig. 3f).

### Identifying the nucleus of paraffin-embedded sections using gold chloride and the NanoSuit method

**Fig. 4:**
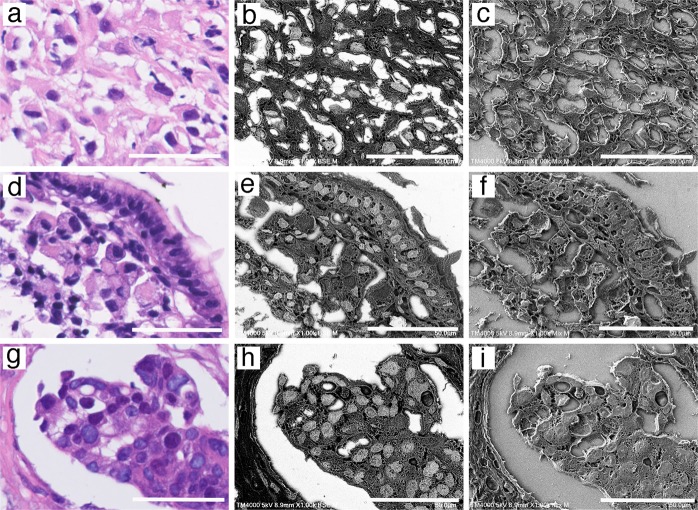
Identification of the nucleus in paraffin sections with gold (III) chloride using the NanoSuit method. Microscopic examination of: **a**–**c** malignant mesothelioma sample, **d**–**f** gastric carcinoma sample (signet ring cell carcinoma), and **g**–**i** breast cancer sample. The panels represent: hematoxylin and eosin (H&E) staining (**a**, **d**, **g**), Low-vacuum scanning electron microscopy (Lv-SEM) image of a sample stained using 1% gold (III) chloride, taken in BSE mode (**b**, **e**, **h**), and mixed Lv-SEM image of a sample stained with 1% gold (III) chloride (**c**, **f**, **i**). The scale bars represent 50 μm

Gold (III) chloride was used as an SEM nuclear staining marker to identify the nucleus of paraffin-embedded sections. To our knowledge, no previous reports have described a nuclear staining protocol for SEM using gold chloride. H&E-stained sections of malignant mesothelioma (Fig. 4a), gastric carcinoma (signet ring cell carcinoma; Fig. 4d), and breast cancer (Fig. 4g) were stained using 1% gold (III) chloride, and the sections faithfully revealed the size and location of the nucleus in light gray when observed by SEM in BSE mode (Fig. 4b, e, h). Mixed (BSE and SE) mode Lv-SEM images showed the 3D structures of paraffin sections (Fig. 4c, f, i).

### Identifying 3,3′-diaminobenzidine (DAB)-stained regions using the NanoSuit method

**Fig. 5:**
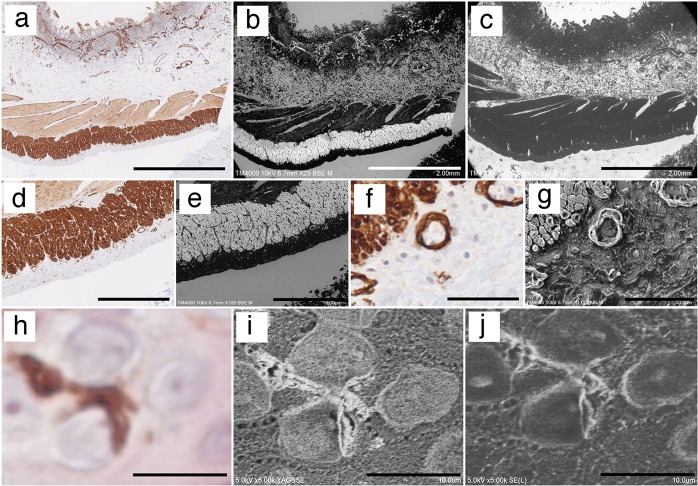
Identifying 3,3′-diaminobenzidine (DAB)-stained regions using the NanoSuit method. **a**–**g** Colon tissue immunostained using an antibody against smooth muscle actin with color development via DAB staining (brown). **b** Staining of the section shown in **a** with 1% gold (III) chloride. The DAB-staining intensity of the low-vacuum scanning electron microscopy (Lv-SEM) image was selectively enhanced as white color, taken in backscattered electron (BSE) mode. **c** Image of a control section without gold (III) chloride treatment. **d** DAB staining of the muscularis propria of a colon tissue section. **e** Lv-SEM image, taken in BSE mode. **f** DAB-stained section of a magnified area of the muscularis propria and blood vessels. **g** Three-dimensional (3D) structure of the correlative region shown in **f**. Lv-SEM image, taken in mixed BSE and SE mode. **h** DAB-stained dendritic cells (DCs) of the human epidermis. **i** Field emission-scanning electron microscopy (FE-SEM) image of DAB-stained DCs after treatment with 1% gold chloride, taken in yttrium–aluminum–garnet BSE mode. **j** 3D structure of DAB-stained DCs in an FE-SEM image, taken in secondary electron mode. The scale bars represent 2 mm (**a**–**c**), 500 (**d**, **e**), 50 (**f**, **g**), and 10 μm (**h**–**j**)

Using the NanoSuit method, conductivity can be maintained without using a heavy metal, and IHC-stained sections are also observable using an SEM. Colon tissue was immunostained using an antibody against smooth muscle actin with the development of DAB coloring (brown; Fig. 5a, d, f). When gold (III) chloride solution was used with DAB-stained sections, DAB-stained regions were selectively enhanced as white areas in Lv-SEM images taken in BSE mode (Fig. 5b, e). No enhancement was seen in DAB-stained areas without gold (III) chloride treatment (Fig. 5c). At a higher magnification, the muscularis propria and blood vessels of the colon (Fig. 5f) were identified as a 3D structure observed in mixed mode (Fig. 5g). Next, human dendritic cells (DCs) in the epidermis were immunostained using a CD1a antibody with DAB (Fig. 5h). Although our light microscope could acquire an obscure magnified DAB-stained image (Fig. 5h), FE-SEM with the NanoSuit method showed a clearer image of DAB-stained DCs after treatment with gold (III) chloride (Fig. 5i, j). These images suggested the possibility that DCs had innervated their processes to the peripheral nucleus. As shown in Fig. 4, nuclear staining was enhanced with gold (III) chloride, but the staining intensity was weaker than the DAB-stained areas obtained in BSE mode (Fig. 5i). Further, the 3D structures of DCs were detected in SE mode (Fig. 5j).

### Direct observation of gold particles in immunostained sections using the NanoSuit method

**Fig. 6:**
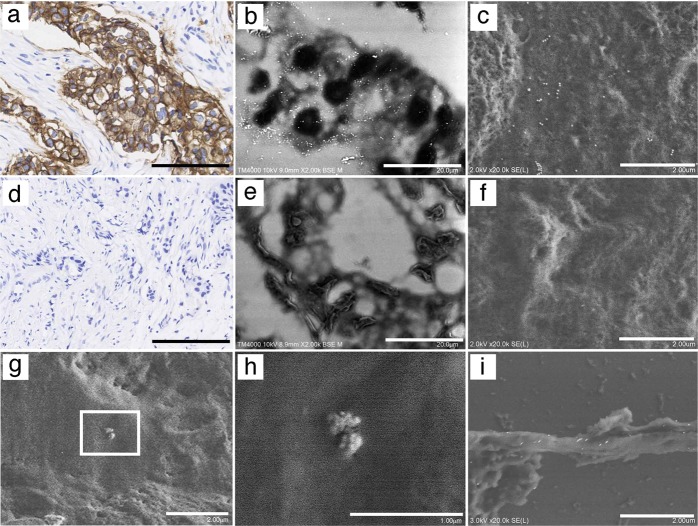
Direct observation of gold particles in immunostained sections using the NanoSuit method. **a**–**c** Staining of a breast cancer section with a human epidermal growth factor receptor 2 (HER2) score of 3+. **a** HER2 expression detected by 3,3′-diaminobenzidine (DAB) staining (brown). HER2 expression observed by detection of 40-nm gold particle signals as multiple white dots in a low-vacuum scanning electron microscopy (Lv-SEM) image taken in backscattered electron (BSE) mode (**b**), or in an FE-SEM taken in secondary electron mode (**c**). Staining of a breast cancer section with a HER2 score of 0. No HER2 expression was detected by DAB staining (brown) (**d**), in an Lv-SEM image taken in BSE mode (**e**), or in an FE-SEM image taken in secondary electron mode (**f**). **g**, **h** Detection of human cytomegalovirus (CMV) with an anti-gB antibody conjugated with 40-nm gold particles. The white square in **g** was magnified and multiple 40-nm gold particles on the surface of human CMV particles are shown in **h**. **i** F-actin detected with 40-nm gold particles on TNT-like structures. The scale bars represent 200 (**a**, **d**), 20 (**b**, **e**), 2 (**c**, **f**, **g**, **i**), and 1 μm (**h**)

First, breast cancer sections were immunostained with an antibody against HER2, and then stained with DAB following the recommended protocol of the HER2 diagnostic kit, with modifications (Fig. 6a, d). Multiple gold particles were detected in breast cancer sections with a HER2 score of 3+ (Fig. 6b), but no gold particles were found in those with a HER2 score of 0 (Fig. 6e) by Lv-SEM in BSE mode. FE-SEM confirmed that 40-nm gold particles were clearly observed in HER2 3+ breast cancer (Fig. 6c), but not in sections with a HER2 score of 0 (Fig. 6f). We applied the same method to identify CMV particles (Fig. 2l). Staining with an antibody against glycoprotein B of human CMV confirmed that virus-like particles were indeed CMV particles (Fig. 6g, h). Moreover, F-actin expression was confirmed on the surface of TNTs (Figs. 3f and 6i).

### EDX analysis of paraffin sections using the NanoSuit method

**Fig. 7:**
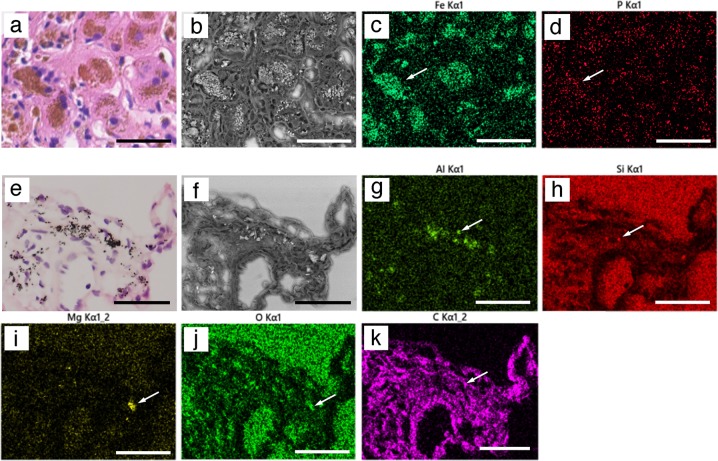
Energy-dispersive X-ray (EDX) analysis of paraffin sections. **a**–**d** Paraffin sections from patients with siderosis. a In hematoxylin and eosin (H&E) sections, putative iron-deposition areas (brown) were identified by light microscopy. **b** A clear backscattered electron (BSE) image (15 kV) was obtained without electric charge. Iron deposition (green, **c**) and phosphorous deposition (red, **d**) were observed by scanning electron microscopy (SEM)/EDX analysis. White arrows show the representative element deposition. **e**–**k** Anthracosis of the lung. With multiple foreign depositions (brown) observed in H&E-stained tissue (**e**). Many of depositions had a strong BSE signal (**f**). Aluminum (green, **g**), silicon (red, **h**), magnesium (yellow, **i**), oxygen (green, **j**), and carbon (purple, **k**) were analyzed by SEM/EDX. The scale bars represent 100 μm

First, H&E sections of patients with siderosis were analyzed. Putative iron-deposition areas were identified in sections after H&E staining (Fig. 7a), and a clear BSE image without an electric charge was taken using the maximum electron beam (15 kV; Fig. 7b). SEM/EDX analysis clearly showed the expected ferrous deposition (Fig. 7c) and phosphorous deposition (Fig. 7d). These data indicated that some of the iron deposits may be comprised of iron phosphide.

Next, anthracosis of the lung was analyzed, and multiple black-to-brown depositions were observed in H&E-stained specimens (Fig. 7e). BSE imaging showed that most depositions had strong backscattered signals, although some of them did not (Fig. 7f), while SEM/EDX analysis revealed the existence of multiple elements (Al, Si, Mg, O, and C) in lung tissue sections (Fig. 7g–k). The pattern of strong BSE signals correlated well with that of aluminum. Collectively, the images shown in Fig. 7f–k reveal that some heavy metals depositions were oxidized.

## Discussion

The number of human tissue specimens stored in biorepositories for clinical purposes was estimated at more than 300 million 15 years ago in the USA alone [11]. If we consider all life science research conducted throughout the world, the number of specimens stored is likely in the billions. Many of these specimens were prepared as FFPE sections for light microscopy, which is a standard clinical and biological observation method that has been used for over a century. In medicine, H&E-stained thin sections on glass slides are usually used for screening purposes to find specific pathological lesions. The samples have been fixed, embedded in wax and subjected to multiple steps of hydration, dehydration, and sometimes antigen retrieval. Formalin fixation is known to induce tissue shrinkage. However, it has been shown that the shrinkage is relatively homogenous and can therefore be corrected for [12]. Nonetheless, there may be a processing artifact caused by paraffin infiltration of large samples not being consistent throughout the sample. While it is often thought that ultrastructural examination of paraffin-embedded tissues is associated with membrane disruption, loss of clarity, and varying degrees of tissue degradation, it has been shown that the ultrastructural study of paraffin-embedded tissue can be optimized by avoiding regions of sections prepared from poorly fixed, or poorly paraffin-filled tissues, and selecting tissue adjacent to necrotic areas [13]. Particulate structures such as neuroendocrine granules and microbes (fungi, protozoa, bacteria, and viruses) are consistently observed by transmission electron microscopy (TEM), even when using paraffin sections [14]. For antigen retrieval, we attempted to use L.A.B solution as much as possible to avoid heat or enzyme damage. Therefore, this study expanded the use of paraffin sections to SEM observations, and the comparison of microstructural changes between “before FFPE processing” and “after FFPE processing” will be awaited as a new project.

SEM typically features three types of detectors, namely SE, BSE, and EDX detectors. SE detectors allow the operator to view the basic topographical structure of a sample by acquiring surface information showing the depth of specimens and mimicking 3D structures. BSE can be utilized to obtain images showing the contrast of different elements present in a sample and EDX provides precise elemental and chemical analysis for a sample. Using these techniques, we could acquire multiple types of information from different perspectives of paraffin sections that were prepared fresh or had been stored.

Recently, super-resolution fluorescence microscopy has been used on formalin-fixed pathological tissues, including human rectal cancers and breast cancer tissue. Furthermore, stimulated emission depletion microscopy (STED) was used to image FFPE human rectal cancer tissues [15], and single molecule localization microscopy (SMLM) was used for breast cancer tissue [16]. Both STED and SMLM use fluorescence to visualize 2D or 3D high-resolution images. Contrastingly, NanoSuit method enables to visualize the high-resolution wet/3D structures of microorganisms and the microstructures of tissues from all kinds of conventional stained sections (H&E- or immunostained-sections) for CLEM. Furthermore, both in principle and on a practical level, resolution of SEM (especially resolution of FE-SEM) is superior to that of super-resolution fluorescence microscopy [17, 18]. We hope that STED and SEM with NanoSuit method will be used together for new discoveries in the future.

After regions of interest are found by light microscopy, an excessive amount of time and effort is usually required to find the same regions of tissues with an SEM due to its high magnification. SSE solution marking at any desired location made it easier to locate the identical pathological region for correlative microscopy between light microscopy and SEM images. Although an organic resistant marker pen can be used to mark tissue sections, it causes coloring and permanently destroys tissues (data not shown). In addition, with the currently developed jig alignment device for CLEM, general-purpose glass slides cannot be used in their current form. Using the NanoSuit method, it was possible to overcome the difficulty of finding the region of interest within a sample on a glass slide. The on-demand droplet spotter system (Fig. 1b) is a useful tool for specifying the exact location of the SSE solution marker without directly contacting the tissue. Further, the thin NanoSuit membrane confers conductivity to the paraffin section and stabilizes the discharge of the NanoSuit solution droplet from the tip of the microcapillary of the on-demand droplet spotter system (data not shown). Conveniently, the SSE solution marker can be removed after SEM observations.

As both biological sections and glass slides are insulators, they were previously covered with metals or carbon for SEM observations [2]. Although the SSE solution contains the elements carbon, hydrogen, oxygen, sodium, and chlorine, SSE drastically reduced the charge effect (Fig. 1f–k). The exact physical mechanism explaining why SSE prevented the charge effects are currently under investigation. Another striking characteristic of SSE is that it caused high water retention inside the tissue under high-vacuum conditions [8, 9]. Thus, an effective thin diffusion barrier [9] combined with SSE solution keeps moisture inside the tissue, which permits the microstructure of paraffin tissues to resemble more closely living tissue structures, such as lung tissues (Fig. 1l), under SEM. Accordingly, the observation of microorganisms, such as fungi, protozoa, bacteria, and viruses (Fig. 2), as well as cytoplasmic vacuolar changes, podocytes, fenestrated vessels of the kidney, skeletal muscle structures, cell projections, TNT-like structures, and microfibrils (Fig. 3) was possible in a 3D/wet form. TNTs are long, actin filament-based, narrow cytoplasmic extensions that are nonadherent when cultured in vitro and are capable of shuttling cellular cargo between connected cells [19, 20]. F-actin is uniformly distributed along the entire length of TNTs; thus it is an important labeling target for TNT-imaging [21]. The presence of CMV (100–300 nm [22]) and TNT-like structures was confirmed by IHC staining using antibodies against CMV-gB and F-actin, respectively (Fig. 6g–i). Future studies may reveal unexpected microstructures by comparing identical light microscopy and SEM images, using the NanoSuit method.

A new staining technique that enables reanalysis of a region of interest by electron microscopy after H&E staining and light microscopy has long been awaited. In addition to observing the structures of cells and tissues with high resolution, it is important to identify the location and shape of the nucleus to discern the states of cells and tissues. The brightness and contrast in an image critically depend on the interaction between the electrons and the sample, and regions with higher atomic numbers in specimens appear brighter than those with lower atomic numbers in an SEM image. Although it is known that uranyl acetate contrasts the nucleus [23], such staining is technically difficult to perform and requires an exhaust device such as a fume hood due to its carcinogenic potential. However, gold (III) chloride staining was found to be a good nuclear marker for SEM using BSE mode in this study (Fig. 4). We also found that gold (III) chloride was useful for immunostaining sections with NanoSuit (Fig. 5). IHC staining is a routine procedure for detecting the expression of biological markers in FFPE tissues. Chromogens, which can appear with different colors (brown, blue, or red) under bright-field microscopy, are localized in fixed tissues to antigens of interest via an antibody-antigen detection system. Similarly, OsO_4_ is known to bind to DAB polymers with high affinity [24, 25]. Newman et al. reported that gold (III) chloride showed high electron density for specific DAB staining, followed closely by OsO_4_. Notably, greater background staining was observed when OsO_4_ was used [26]. While a conventional coating technique can be used to represent an SEM image, the conductivity of the thin NanoSuit membrane obviates the need to sputter heavy metals. In addition to the conductivity, the electric beam can penetrate the specimen to some extent through the NanoSuit membrane, which allows for the location of gold elements to be determined with high contrast.

Further, the technique allowed the observation of the processes of CD1a-positive DCs to have innervated the peripheral rim of the nucleus of a neighboring epidermal cell (Fig. 5h–j). Epidermal DCs are star-shaped, wandering macrophages derived from the bone marrow that infiltrate the epidermal layers and serve the immune functions of phagocytosis and antigen presentation. It is believed that DC processes extend between epidermal squamous cells [27]. Our results indicated the possibility that there may be an interaction at the nuclear level. The reasons why innervation of DC processes to the peripheral rim of the nucleus has not been reported may be as follows; light microscopy may not have sufficient magnification for DAB-stained DCs, conventional SEM methods may fail in visualizing cross-sectional views, and TEM sections may be too thin to find this structure. However, we successfully identified DAB-stained DCs using the NanoSuit method. The idea that DCs might directly exchange information with the nucleus is a revolutionary proposal. Further study will be needed to define the detail structures by TEM and the function of this structure in detail.

Determining target protein-expression levels in tissues is important for understanding biological and disease-associated mechanisms, leading to molecular target-based drug therapies. Without the use of a metal coating, the NanoSuit method can be applied for quantitative diagnostic imaging of cancer tissue using gold particles. Further, the quantitative sensitivity when IHC staining is developed with DAB (IHC-DAB) is low [28]. In IHC with DAB staining (IHC-DAB), the intensity of DAB staining depends on the enzymatic activity of horseradish peroxidase (HRP) and is significantly affected by the reaction time, temperature, and HRP substrate concentration. Although fluorescent IHC staining can effectively increase the quantitative sensitivity of conventional IHC staining, tissue autofluorescence is detrimental to the sensitivity. To improve quantitation, Gonda et al. developed new fluorescent nanoparticles called phosphor-integrated dots, which are brighter and have a wider dynamic range than commercially available fluorescent nanoparticles, such as quantum dots [28]. Our results showing that we could count the metal particles directly using the NanoSuit method in combination with SEM indicated another possibility for quantitative diagnostic in the future.

Finally, the application of the NanoSuit method to EDX may allow for the reanalysis of the many FFPE tissues stored throughout the world. Many researchers often need to identify areas suspected of having a foreign deposition in H&E sections. Therefore, special staining for each foreign deposition is chosen for elemental analysis. However, this staining method is labor-intensive and requires substantial experience. Scimeca et al. [29] reported that EDX microanalysis could represent a powerful tool (i) to investigate the accumulation of heavy metals in tissues and in forensic science, (ii) to identify specific asbestos isotypes for linking the pulmonary asbestos-related disease to previous workplace exposure, (iii) to characterize different calcification isotypes and obtain additional information regarding the link between calcification and disease, and (iv) to study the toxic effects of nanoparticles and their potential for drug delivery. Thus, a new TEM-EDX analytical protocol may be helpful for detecting elements, asbestos nanofibers, and nanomaterials in general when working with archival FFPE tissues. Previously, H&E-stained sections were used to identify areas to be subjected to EDX analysis [30]. However, this method destroys the specimens permanently, and sample handling is difficult. For conventional SEM-EDX analysis, specimens should be carefully treated. To prevent cell surface deformation, tissues that undergo SEM analysis are dehydrated using critical-point drying instruments with supercritical CO_2_. However, when using these analytical methods, diffusible atoms, elements, and small molecules are easily lost and/or move during the dehydration and embedding procedures. To prevent these effects, cryofixation techniques have been explored, but only a few groups are using this approach because of the necessity for special tools and the difficulty of the method [29]. We successfully analyzed the elements in the correlative region of H&E sections using the NanoSuit method. NanoSuit could protect H&E paraffin sections against drying and could preserve elements and small molecules for EDX analysis. By imparting electrical conductivity to the section on the glass slide with the SSE solution, it was possible to analyze the elements of the identical H&E-stained specimen for the first-time using SEM/EDX. The background of silicon and oxygen signals on the glass cannot be ignored, but was clearly discriminated from other elements. Carbon is a basic component of organic tissue and was fairly evenly distributed in the lung tissue. The carbon contained in the NanoSuit coating on glass slides was not detectable (Fig. 7k). Therefore, the NanoSuit coating hardly affected carbon analysis.

Table 1 summarizes the results of this study. Briefly, both light and scanning electron microscopes can be used easily and accurately at low cost with the NanoSuit method. We successfully observed the 3D structures of pathogens, including CMV particles, and indicated the innervation of processes of DCs to the adjacent cell’s nucleus rim in the paraffin sections. Because the NanoSuit method prevents retention of electrons on the specimen even when located on an insulating glass slide, it was possible to obtain both clear images and conduct elemental analysis. In addition, SSE solution can be removed after observation. Therefore, important glass slides can be restained and stored. In conclusion, the NanoSuit method for CLEM should open new approaches for histological examination, leading to unexpected discoveries, and will also contribute to medical practice.

**Table 1:** Items that become possible using the NanoSuit method with paraffin sections

Items become possible by NanoSuit method for paraffin sections
Easy correlative observation of light microscopic and SEM observation for commonly used glass slides
Restoring to original H&E color after SEM observation
Observation of wet/3D structure of microorganisms with high magnification
Observation of wet/3D microstructures
Identification of nucleus by gold chloride
Identification of DAB-stained area by gold chloride
Possibility of quantitative immunohistochemical diagnosis using gold particles
Analysis of elements of paraffin sections on glass slides

*SEM* scanning electron microscopy, *H&E* hematoxylin and eosin, *3D* three-dimensional, *DAB* 3,3′-diaminobenzidine
